# Threefold helical assembly *via* hy­droxy hydrogen bonds: the 2:1 co-crystal of bi­cyclo­[3.3.0]octane-*endo*-3,*endo*-7-diol and bi­cyclo­[3.3.0]octane-*endo*-3,*exo*-7-diol

**DOI:** 10.1107/S2056989021001730

**Published:** 2021-02-19

**Authors:** Isa Y. H. Chan, Mohan M. Bhadbhade, Roger Bishop

**Affiliations:** a School of Chemistry, University of New South Wales, UNSW Sydney NSW 2052, Australia; b Mark Wainwright Analytical Centre, University of New South Wales, UNSW Sydney, NSW 2052, Australia

**Keywords:** co-crystal, failure of fractional recrystallization, crystal structure, hydrogen-bonded complex, isomers, complementarity, pseudo-threefold screw axis.

## Abstract

Reduction of bi­cyclo­[3.3.0]octane-3,7-dione yields a mixture of the *endo*-3,*endo*-7-diol and *endo*-3, *exo*-7-diol (C_8_H_14_O_2_) isomers (**5** and **6**). These form (**5**)_2_·(**6**) co-crystals in the monoclinic *P*2_1_/*n* space group (with *Z* = 6, *Z*′ = 1.5) rather than undergoing separation by means of fractional recrystallization or column chromatography. The mol­ecule of **5** occupies a general position, whereas the mol­ecule of **6** is disordered over two orientations across a centre of symmetry with occupancies of 0.463 (2) and 0.037 (2). Individual diol hy­droxy groups associate around a pseudo-threefold screw axis by means of hydrogen bonding. The second hy­droxy group of each diol behaves in a similar manner, generating a three-dimensional hydrogen-bonded network structure. This hydrogen-bond connectivity is identical to that present in three known helical tubuland diol–hydro­quinone co-crystals, and the new crystal structure is even more similar to two homologous aliphatic diol co-crystals.

## Abstract 2

The structure of bi­cyclo­[3.3.0]octane-*endo*-3,*endo*-7-diol and bi­cyclo­[3.3.0]octane-*endo*-3,*exo*-7-diol, C_8_H_14_O_2_ form 2:1 co-crystals in the monoclinic *P*2_1_/*n* space group rather than undergoing separation by means of fractional recrystallization or column chromatography.

## Chemical context   

Crystalline binary adducts (Herbstein, 2005 ▸) have been classified as clathrates, coordinatoclathrates, clathratocomplexes or complexes (Weber & Josel, 1983 ▸). At one end of this structural continuum, clathrates have a dominant host structure, host–guest inter­actions are less important, and the guests are spatially caged. Complexes, on the other hand, are mutually coordinated and the importance of three-dimensional enclosure is significantly lessened. Hosts may complex with a liquid guest to yield solvates or hydrates. If the two components are both solids of comparable size, however, then the host–guest distinction vanishes. The latter group of complexes are nowadays generally termed co-crystals (Aakeröy & Chopade, 2012 ▸).

Research into co-crystals is an area of considerable current significance. Many potentially valuable bioactive mol­ecules have poor aqueous solubility and this restricts their application as pharmaceutical drugs. Combination with a benign partner mol­ecule to produce a co-crystal can result in enhanced properties such as improved drug formulation and greater biological uptake (Almarsson & Zaworotko, 2004 ▸). Our knowledge of inter­molecular attractive forces often allows a prediction to be made of the complementary partner required for such pharmaceutical co-crystal synthesis (Bis *et al.*, 2006 ▸, 2007 ▸).

**Figure 1:**
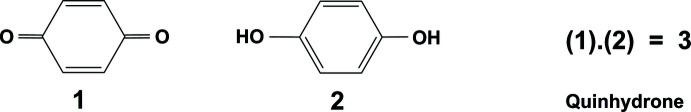
1:1 combination of *p*-benzo­quinone **1** and hydro­quinone **2**, commonly known as quinhydrone **3**

A second sub-set of co-crystalline substances comprises unexpected combinations of isomers or structurally related compounds (Kelley *et al.*, 2011 ▸). This is not a new phenomenon. Indeed, the first such material now recognised as being a co-crystal was discovered in 1844 by Friedrich Wöhler. This was the 1:1 combination of *p*-benzo­quinone **1** and hydro­quinone **2**, commonly known as quinhydrone **3** (Fig. 1 ▸) (Karagianni *et al.*, 2018 ▸; Sakurai, 1968 ▸). These novel co-crystalline materials are generally discovered accidentally as a consequence of preparative organic work going wrong, in particular the very few instances where standard purification techniques fail. It is therefore a rare and unpredictable occurrence.

**Figure 2:**
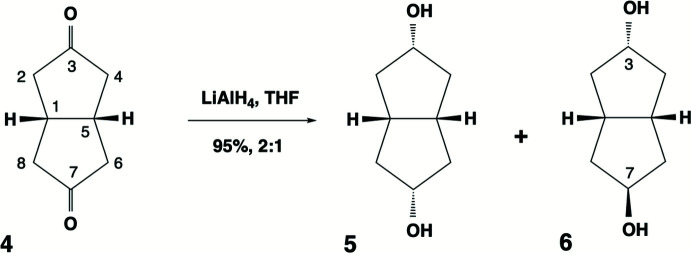
Synthetic route to formation of title compounds **5** and **6**

The present work describes a new example of this phenomenon. Reduction of bi­cyclo­[3.3.0]octane-3,7-dione **4** with lithium aluminium hydride yielded an approximately 2:1 mixture of the diols **5** and **6** (Fig. 2 ▸). These isomeric products could not be separated by fractional recrystallization or standard column chromatography using silica or alumina.
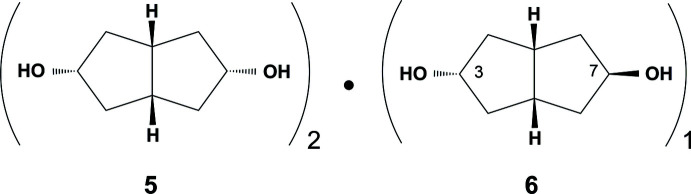


## Structural commentary   

**Figure 3:**
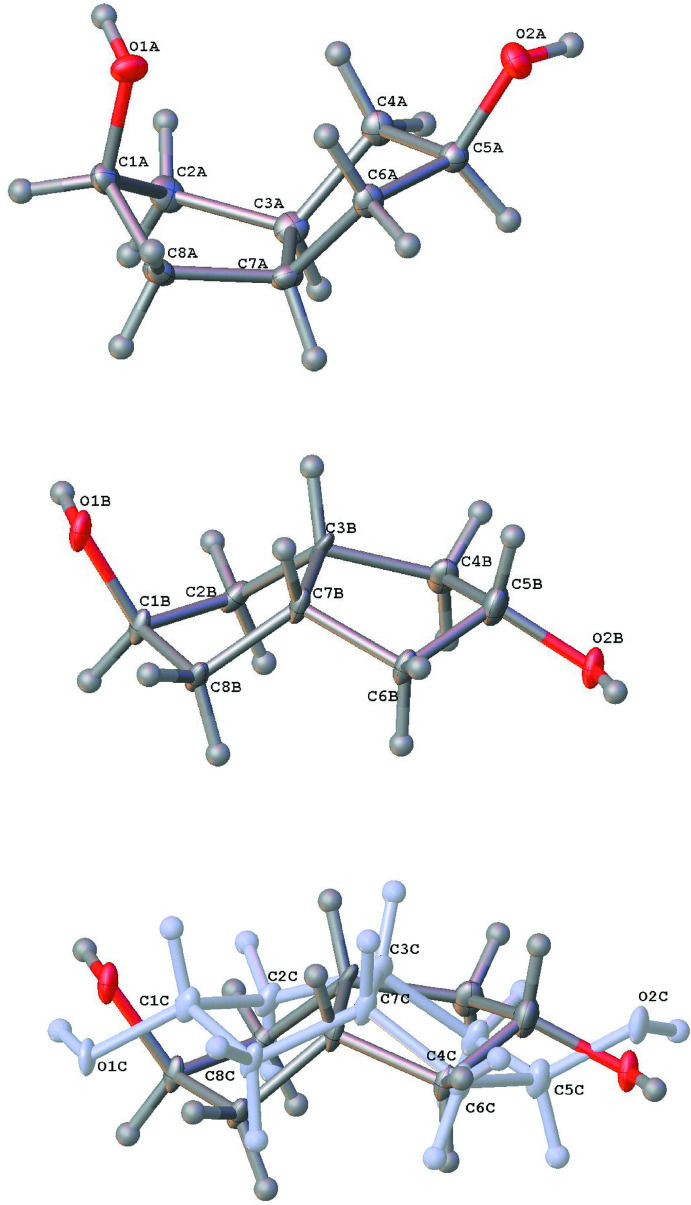
The isomer **5** (mol­ecule *A*) (upper), the isomer **6** with its major component (mol­ecule *B*, centre) and minor components (mol­ecule *C*, lower) showing their crystallographic atom labelling. Displacement ellipsoids are drawn at the 50% probability level and hydrogen atoms are shown as spheres of arbitrary size.

When the mixture of diols **5** and **6** was recrystallized from toluene, thin plate crystals of composition (**5**)_2_·(**6**) were produced in the monoclinic space group *P*2_1_/*n*. The mol­ecules of **5** (atoms labelled with suffix *A*) are in a general position, whereas mol­ecules of **6** are disordered across the centre of inversion in this space group (thus *Z* = 6, *Z*′ = 1.5). There are two sites (atoms labelled with suffixes *B* and *C*) of occupancies 0.463 (2) and 0.037 (2), respectively. The minor site *C* can be described as a position obtained by a twofold rotation about the axis perpendicular to the midpoint of the bond C3*B*—C7*B* of the mol­ecule at site *B* (Fig. 3 ▸). However, from here onwards, only the major site *B* will be used in figures and discussions of inter­molecular inter­actions for the sake of clarity. The bi­cyclo­[3.3.0]octane skeleton comprises two cyclo­pentane rings fused in a *cis*-manner. Its basic configuration is a flattened V-shape in solution, with the convex face being termed *exo*- and the concave face *endo*-. Reduction of the diketone **4** therefore occurs preferentially on the more exposed *exo*-face to produce the *endo*-alcohol configuration. The co-crystal formula indicates that this stereoselectivity is around 5:1 using lithium aluminium hydride in tetra­hydro­furan.

**Figure 4:**
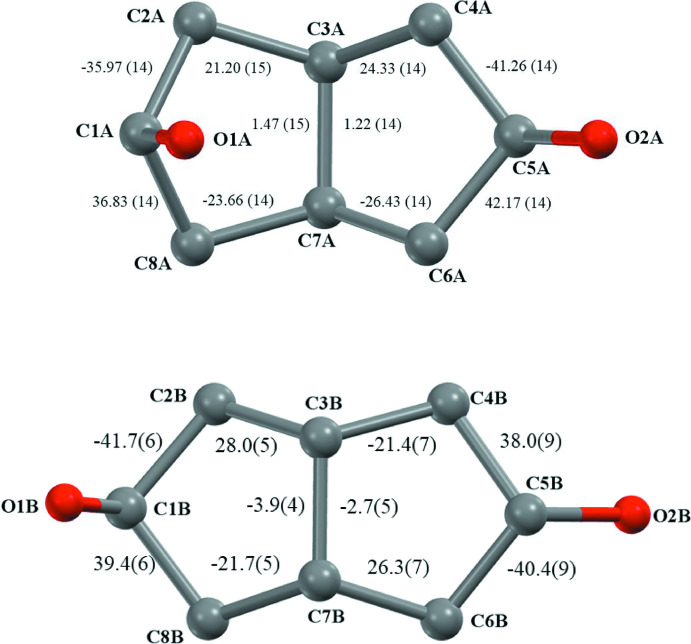
The bi­cyclo­[3.3.0]octane ring conformations adopted by the isomers **5** (upper) and **6** (lower) in the structure (**5**)_2_·(**6**). Torsion angles are shown with their e.s.d.s.

The cyclo­pentane rings, however, have conformational mobility that can contribute to the optimal crystal packing. In particular, envelope conformations may occur with the envelope flap being orientated *syn*- to either of the *exo*- or *endo*-faces. The isomer **5** has one flap *syn* to each of these ring faces, while **6** has both its flaps *syn* to the *exo*-face of the structure (Fig. 3 ▸). Ring twisting can also occur but is relatively minor in (**5**)_2_·(**6**). Qu­anti­tative descriptions of these conformational effects are summarized by the cyclo­pentane ring torsion angle values marked on Fig. 4 ▸.

## Supra­molecular features   

**Figure 5:**
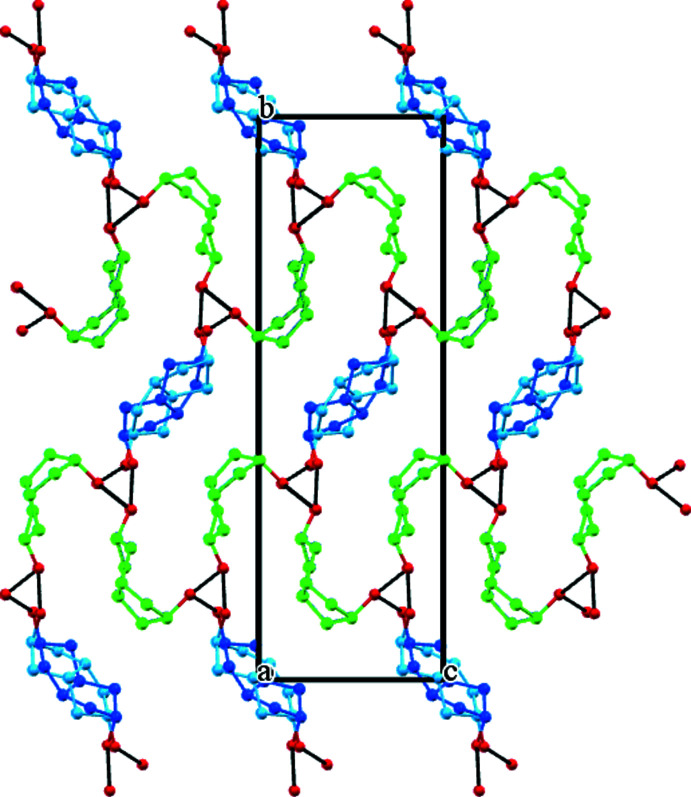
The crystal structure of (**5**)_2_·(**6**) projected on the *bc* plane and looking down the pseudo-threefold screw axes. Colour code: O atoms red, diol **5** green, and diol **6** major component (light and dark blue). Minor component C and all hydrogen atoms are omitted for clarity and the hy­droxy hydrogen bonds are indicated as solid black lines.

**Figure 6:**
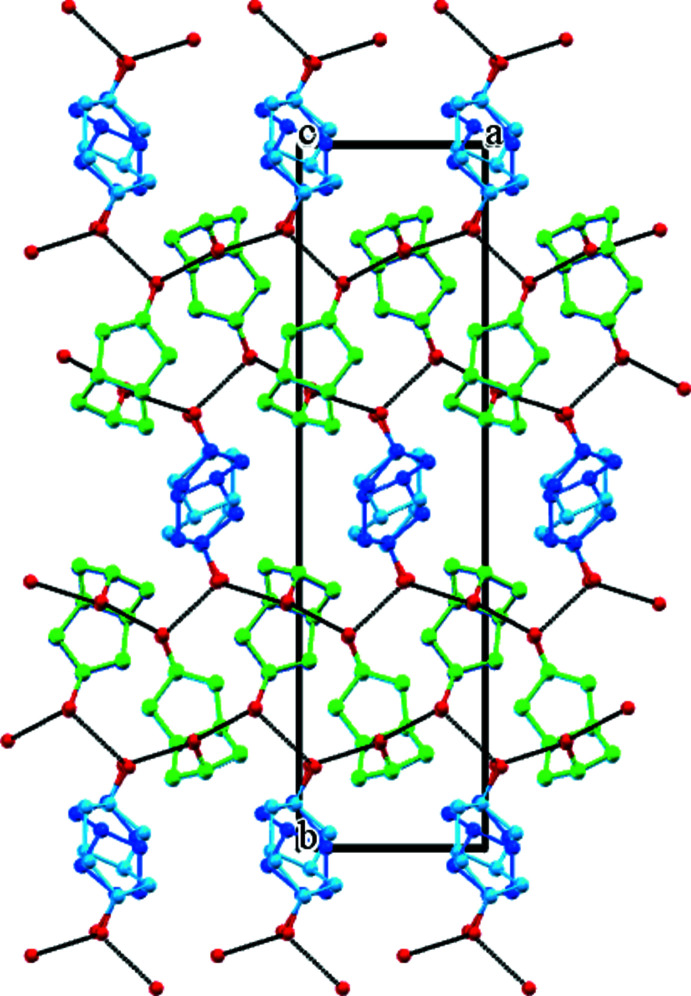
The crystal structure of (**5**)_2_·(**6**) projected on the *ab* plane and showing the pseudo-threefold screw axes running horizontally. The alternating zones of isomers **5** and **6** in the crystal should be noted. Colour code is the same as used in Fig. 5 ▸.

The isomeric diol mol­ecules are connected by hy­droxy hydrogen bonds (Table 1 ▸) and a three-dimensional network is formed. Mol­ecules of **5** and **6** form a 2:1 infinite chain with their hydrogen bonds surrounding a pseudo-threefold screw axis along the *a*-axis direction (Fig. 5 ▸). The O⋯O distance between mol­ecules of **5** is 2.743 (2) Å, and those between **5** and **6** are 2.629 (12) and 2.784 (12) Å. Mol­ecules of **6** are not connected directly through hydrogen bonding with each other. Both the second hy­droxy groups of **5** and **6** contribute to further identical screw axis assemblies. Hence the resulting crystal contains alternating zones of **5** and **6** mol­ecules that run along both the *a*- and *c*-axis directions (Fig. 6 ▸). The only other notable inter­action is a C7*A*—H7*A*⋯O2*A* weak hydrogen bond [*D* 3.727 (2), *d* 2.80 Å] that links adjacent mol­ecules of **5**.

Viewed down *a*, the isomer **5** is present as two stacked columns of translated diol mol­ecules. It is therefore probable that diol **6** is stacked similarly. This isomer contains no centre of symmetry, but is situated on a crystallographic inversion site. Mol­ecules of **6** therefore appear in Figs. 5 ▸ and 6 ▸ as a superimposition of two disordered forms across a centre of symmetry.

**Figure 7:**
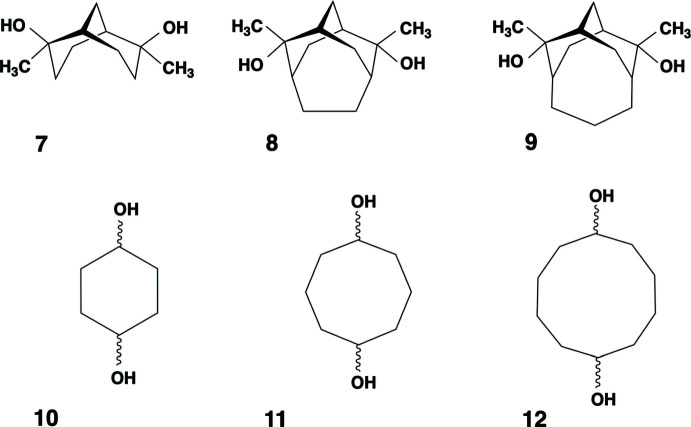
Mol­ecular structures of diols **7**–**10** and **12** that can be used as co-crystal partners to generate the same hydrogen bonding connectivity as that present in (**5**)_2_·(**6**). We predict that cyclo­octane-1,5-diol **11** will behave similarly.

**Figure 8:**
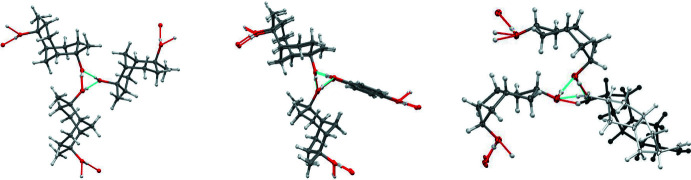
Comparison of the threefold screw axis of crystalline **7** (left), and the pseudo-threefold screw axes present in the co-crystals (**7**)_2_·(**2**) (centre) and (**5**)_2_·(**6**) (right).

The hy­droxy hydrogen-bonding connectivity present in (**5**)_2_·(**6**) provides a versatile supra­molecular network that occurs in at least five other diol co-crystal structures (Fig. 7 ▸). Helical tubuland (HT) diols **7**–**9** employ hy­droxy group hydrogen bonding to assemble around threefold screw axes in space group *P*3_1_21 (Bishop, 2009 ▸). This creates tubular voids that enclose guest mol­ecules of many structural types. A notable exception is the phenol family, which instead yields hydrogen-bonded co-crystals. This is achieved by one of the three columns of HT diol mol­ecules being replaced by a column of phenols with concomitant formation of pseudo-threefold screw axes. Co-crystals of general formula (**HT**)_2_·(**2**) are produced when hydro­quinone **2** is used as the co-former mol­ecule (Ung *et al.*, 1993 ▸, 1994 ▸; Yue *et al.*, 2002 ▸). Fig. 8 ▸ compares the threefold and pseudo-threefold screw axes using the example of HT diol **7**. These should be compared to the screw axis observed in (**5**)_2_·(**6**) (Fig. 8 ▸, right).

**Figure 9:**
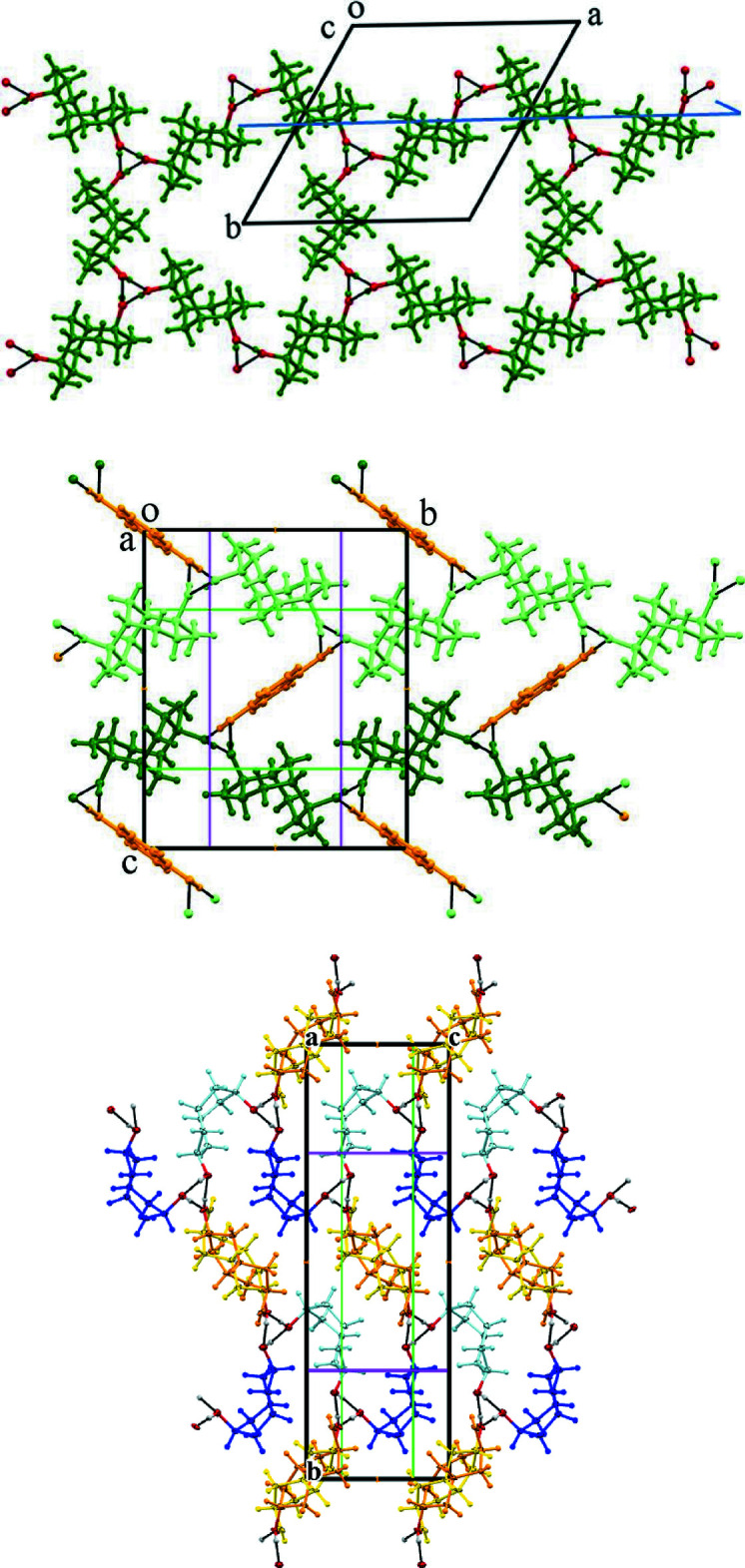
Comparison of the hydrogen-bonded networks present in crystals of **7** (upper), (**7**)_2_·(**2**) (centre), and (**5**)_2_·(**6**) (lower) and looking down the threefold screw axes. Only one enanti­omer (dark green) is present in structure **7**, but both enanti­omers (light and dark green) are present in compound (**7**)_2_·(**2**). Only achiral mol­ecules are contained in (**5**)_2_·(**6**). The mol­ecules of isomer **5** (light and dark blue) occupy a general position, whereas those of isomer **6** major component (yellow and orange) are equally distributed across a centre of symmetry.

The hydrogen-bonding networks of **7**, (**7**)_2_·(**2**), and (**5**)_2_·(**6**) are compared in Fig. 9 ▸ (upper, centre, and lower). All are viewed looking down the threefold screw axes. Despite the very different shapes and mol­ecular structures of the building blocks **7**/**5** and **2**/**6**, their hy­droxy hydrogen-bonding connectivity is identical. The three networks do, however, differ in their crystallographic symmetry. This is a consequence of the presence, or absence, of chirality.

Structure **7** in chiral space group *P*3_1_21 contains only one enanti­omer (dark green), and the threefold hy­droxy hydrogen bonding coincides with the crystallographic 3_1_ screw axis. Mol­ecules along *b* surround a 2_1_ screw axis (blue line), but mirror (or glide) symmetry is absent.

Crystallization of the racemic HT diol and hydro­quinone yields the co-crystal (**7**)_2_·(**2**) in space group *P*2_1_/*c*. Diol mol­ecules of opposite chirality (light or dark green) are separated in the crystal, with each enanti­omer forming an infinite chain around a 2_1_ screw axis running along *b* (green lines). Adjacent chains are bridged by achiral hydro­quinone guests (orange), the inversion centre of which coincides with the crystallographic centre of symmetry. The enanti­omeric diol chains are related by a *c*-glide (magenta lines). Hydro­quinone mol­ecules link the HT diol chains by contributing their hy­droxy groups for completion of the pseudo-threefold hydrogen-bonded helices running along *a*.

In contrast, both the diol mol­ecules forming compound (**5**)_2_·(**6**) are achiral, but this present case in space group *P*2_1_/*n* reveals a further example of threefold helicity involving different symmetry elements. All the mol­ecules of isomer **5** are identical, but here have been coloured light or dark blue to discriminate those related by mirror symmetry operation. The second diol isomer **6** is shown in yellow and orange.

The hy­droxy groups of both isomers associate to produce hydrogen-bonded pseudo-threefold helices down *c*. Mol­ecules of **5** surround a 2_1_ screw axis running along *b* (green lines), but are not connected by hydrogen bonds. They are also arranged as chains in the *c*-axis direction and these chains are related by a *c*-glide (magenta lines). The isomer **6** performs the same roles as hydro­quinone did in the previous structure. These bridging mol­ecules are located at the crystallographic inversion centre but lack their own centre of symmetry. Hence there is disorder of isomer **6** that creates a statistical centre of symmetry.

## Database survey   

The reduction of dione **4** using sodium borohydride or samarium iodide was earlier investigated by Camps *et al.*, (2001 ▸). Small amounts of the pure isomers **5** and **6** were isolated, and these compounds were fully characterized by IR, ^1^H and ^13^C NMR, MS, and combustion analysis. No indication of mol­ecular inclusion was evident. X-ray structure determin­ations of these pure isomers are unreported.

Kelley *et al.* (2011 ▸) have carried out a comprehensive survey titled *Failures of fractional recrystallization: ordered co-crystals of isomers and near isomers*. This ground-breaking database search revealed 270 X-ray determinations of ordered co-crystals between isomers or closely related compounds. The phenomenon has therefore been demonstrated to be extremely rare. It will occur where the two partner mol­ecules share structural complementarity and near identical solubility. New examples of this phenomenon cannot usually be predicted.

However, we note that cyclo­hexane-1,4-diol **10** (Loehlin *et al.*, 2008 ▸) and cyclo­decane-1,6-diol **12** (Ermer *et al.*, 1989 ▸) both form 2:1 *cis*:*trans* diol co-crystals that are extremely similar to our compound (**5**)_2_·(**6**). These three examples share a simple mol­ecular structure in which two secondary alcohol groups are connected, maintaining net mirror plane symmetry, by means of a cyclic aliphatic linking group. This suggests that other members of this family exist. A probable example is cyclo­octane-1,5-diol **11** but, at present, only the X-ray structure of its *cis*-isomer has been reported (Miller & McPhail, 1979 ▸).

## Synthesis and crystallization   

A solution of bi­cyclo­[3.3.0]octane-3,7-dione **4** (0.40 g, 2.90 mmol) in dry tetra­hydro­furan (THF, 15 mL) was added dropwise to a stirred ice-cooled solution of lithium aluminium hydride (0.28 g, 7.38 mmol) in dry THF (15 mL). The ice-bath was removed once addition was complete and the mixture stirred overnight at room temperature. Excess LiAlH_4_ was decomposed by cautious addition of wet diethyl ether. Saturated ammonium chloride solution was then added and the reaction product extracted five times using ethyl acetate. The combined organic extracts were dried (anhydrous Na_2_SO_4_), filtered, and the solvents evaporated to yield a 2:1 mixture of the stereoisomers **5** and **6** (0.39 g, 95%). ^1^H NMR (300 MHz, CDCl_3_) δ, combined for **5/6**: 1.20–1.29 (*m*, 2H), 1.62–1.71 (*m*, 4H), 2.01–2.11 (*m*, 4H), 2.47–2.54 (*m*, 2H), 4.07–4.12 (*m*, 1H), 4.18–4.25 (*m*, 1H); ^13^C NMR (75.4 MHz, CDCl_3_) δ, for **5**: 41.3 (CH), 43.4 (CH_2_), 76.3 (CH); for **6**: 38.5 (CH), 42.4 (CH_2_), 42.6 (CH_2_), 74.6 (C), 75.1 (C). The diol isomers could not be separated by means of column chromatography (silica gel 60 230-400 mesh, neutral or basic aluminium oxide 150 mesh Brockmann activity 1). Attempted crystallization of the diol mixture from benzene, 1,4-dioxane, ethanol, or ethyl acetate did not give crystalline material. Thin needle-like crystals were produced from a cyclo­hexane solution, and thin plates were obtained from di­chloro­methane or toluene. The latter were of sufficient quality for single crystal X-ray structure determ­ination at the Australian Synchrotron facility.

## Refinement   

Crystal data, data collection and structure refinement details are summarized in Table 2 ▸. The mol­ecule of **6** (suffix *B*) is located on a crystallographic inversion centre, which is incompatible with the mol­ecular symmetry of the mol­ecule. The mol­ecule was thus refined as 1:1 disordered across this inversion centre. Close inspection of the difference densities revealed additional disorder, by an approximate twofold rotation perpendicular to the C3—C7 bond, and a second minor disordered moiety was added to the refinement model (suffix *C*). Bond distances and angles of both disordered moieties were restrained to be similar to that of the ordered mol­ecule of **5** (suffix *A*) using a *SHELXL* SAME command (the esd used was 0.02 Å). *U*_ij_ components of ADPs of disordered atoms were restrained to be similar for atoms closer to each other than 2.0 Å using a *SHELXL* SIMU command (the esd used was 0.01 Å^2^). The atom O1*B* and the symmetry equivalent (by inversion) of O2*B* occupy nearly identical positions, and their ADPs were constrained to be identical (*SHELXL* command EADP). Subject to these conditions, the occupancy rates refined to two times 0.463 (2) (moiety *B* and its inversion-created counterpart) and two times 0.037 (2) (moiety *C* and its inversion-created counterpart).

The minor moiety hy­droxy atoms (of *C*) were in addition restrained based on hydrogen-bonding considerations. H2*C* was restrained to have a distance of 1.90 (2) Å from O2*A* (at 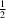 + *x*, 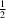 − *y*, −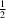 + *z*), and H1*CA* to have a distance of 2.05 (2) Å from O2*A* (at 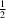 − *x*, 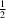 + *y*, 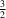 − *z*).

Most of the H atoms (except for minor disordered component C) could be located in difference maps and the remaining were fixed at stereochemically reasonable positions using appropriate AFIX commands. In the final structural model, all H atoms were treated as riding atoms in geometrically idealized positions, with C—H distances of 0.99 Å (CH_2_) and 0.84 Å (OH), and with *U*_iso_(H) = *kU*_eq_(C), where *k* = 1.5 for OH groups, and 1.2 for all other H atoms.

## Supplementary Material

Crystal structure: contains datablock(s) I. DOI: 10.1107/S2056989021001730/zl2800sup1.cif

Supporting information file. DOI: 10.1107/S2056989021001730/zl2800Isup3.cml

Structure factors: contains datablock(s) I. DOI: 10.1107/S2056989021001730/zl2800Isup3.hkl

CCDC reference: 2019203

Additional supporting information:  crystallographic information; 3D view; checkCIF report

## Figures and Tables

**Table 1:** Hydrogen-bond geometry (Å, °)

*D*—H⋯*A*	*D*—H	H⋯*A*	*D*⋯*A*	*D*—H⋯*A*
O1*A*—H1*A*⋯O1*B* ^i^	0.84	1.89	2.732 (12)	177
O1*A*—H1*A*⋯O2*B* ^ii^	0.84	1.88	2.710 (12)	170
O1*A*—H1*A*⋯O1*C* ^i^	0.84	2.05	2.83 (6)	154
O1*A*—H1*A*⋯O2*C* ^ii^	0.84	2.04	2.82 (7)	155
C2*A*—H2*AA*⋯O1*C* ^i^	0.99	2.37	3.18 (5)	139
C2*A*—H2*AA*⋯O2*C* ^ii^	0.99	2.44	3.25 (6)	139
O2*A*—H2*A*⋯O1*A* ^iii^	0.84	1.91	2.7432 (16)	173
O1*B*—H1*B*⋯O2*A* ^iv^	0.84	1.98	2.784 (12)	159
O2*B*—H2*B*⋯O2*A* ^iii^	0.84	1.79	2.629 (12)	173
O1*C*—H1*C*⋯O2*A* ^iv^	0.84	2.04	2.86 (5)	164

Symmetry codes: (i) -x+1, -y+1, -z+2; (ii) x, y, z+1; (iii) x+{\script{1\over 2}}, -y+{\script{1\over 2}}, z-{\script{1\over 2}}; (iv) -x+{\script{1\over 2}}, y+{\script{1\over 2}}, -z+{\script{3\over 2}}.

**Table 2:** Experimental details

Crystal data	
Chemical formula	0.66C_8_H_14_O_2_·0.33C_8_H_14_O_2_
*M*_r_	142.19
Crystal system, space group	Monoclinic, *P*2_1_/*n*
Temperature (K)	100
*a*, *b*, *c* (Å)	6.2758 (3), 23.6912 (10), 7.776 (4)
β (°)	91.21 (2)
*V* (Å^3^)	1155.9 (6)
*Z*	6
Radiation type	Synchrotron, λ = 0.71073 Å
μ (mm^−1^)	0.09
Crystal size (mm)	0.02 × 0.02 × 0.01

Data collection	
Diffractometer	Area detector at Australian Synchrotron
No. of measured, independent and observed [*I* > 2σ(*I*)] reflections	16006, 2215, 2021
*R*_int_	0.066
(sin θ/λ)_max_ (Å^−1^)	0.617

Refinement	
*R*[*F* ^2^ > 2σ(*F* ^2^)], *wR*(*F* ^2^), *S*	0.049, 0.127, 1.06
No. of reflections	2215
No. of parameters	272
No. of restraints	399
H-atom treatment	H-atom parameters constrained
Δρ_max_, Δρ_min_ (e Å^−3^)	0.35, −0.23

Computer programs: *XDS* (Kabsch, 2010 ▸), *SHELXT2018/2* (Sheldrick, 2015*a* ▸), *SHELXL* (Sheldrick, 2015*b* ▸) and *OLEX2* (Dolomanov *et al.*, 2009 ▸).

## supplementary crystallographic information

### Crystal data

**Table:**

0.66C_8_H_14_O_2_·0.33C_8_H_14_O_2_	*F*(000) = 468
*M**_r_* = 142.19	*D*_x_ = 1.226 Mg m^−^^3^
Monoclinic, *P*2_1_/*n*	Synchrotron radiation, λ = 0.71073 Å
*a* = 6.2758 (3) Å	Cell parameters from 1189 reflections
*b* = 23.6912 (10) Å	θ = 1–22°
*c* = 7.776 (4) Å	µ = 0.09 mm^−^^1^
β = 91.21 (2)°	*T* = 100 K
*V* = 1155.9 (6) Å^3^	Plate, colourless
*Z* = 6	0.02 × 0.02 × 0.01 mm

### Data collection

**Table:**

Area detector at Australian Synchrotron diffractometer	*R*_int_ = 0.066
phi scan	θ_max_ = 26.0°, θ_min_ = 3.4°
16006 measured reflections	*h* = −7→7
2215 independent reflections	*k* = −29→29
2021 reflections with *I* > 2σ(*I*)	*l* = −9→9

### Refinement

**Table:**

Refinement on *F*^2^	Primary atom site location: dual
Least-squares matrix: full	Secondary atom site location: difference Fourier map
*R*[*F*^2^ > 2σ(*F*^2^)] = 0.049	Hydrogen site location: inferred from neighbouring sites
*wR*(*F*^2^) = 0.127	H-atom parameters constrained
*S* = 1.06	*w* = 1/[σ^2^(*F*_o_^2^) + (0.0636*P*)^2^ + 0.7974*P*] where *P* = (*F*_o_^2^ + 2*F*_c_^2^)/3
2215 reflections	(Δ/σ)_max_ = 0.002
272 parameters	Δρ_max_ = 0.35 e Å^−^^3^
399 restraints	Δρ_min_ = −0.23 e Å^−^^3^

### Special details

**Table:**

Geometry. All esds (except the esd in the dihedral angle between two l.s. planes) are estimated using the full covariance matrix. The cell esds are taken into account individually in the estimation of esds in distances, angles and torsion angles; correlations between esds in cell parameters are only used when they are defined by crystal symmetry. An approximate (isotropic) treatment of cell esds is used for estimating esds involving l.s. planes.

### Fractional atomic coordinates and isotropic or equivalent isotropic displacement parameters (Å^2^)

**Table:**

	*x*	*y*	*z*	*U*_iso_*/*U*_eq_	Occ. (<1)
O1A	0.06519 (17)	0.34885 (4)	1.12882 (14)	0.0164 (3)	
H1A	0.172045	0.360542	1.185108	0.025*	
C1A	0.0032 (2)	0.39024 (6)	1.00232 (19)	0.0153 (3)	
H1AA	−0.038281	0.426466	1.058451	0.018*	
C2A	0.1778 (3)	0.40019 (6)	0.8703 (2)	0.0180 (3)	
H2AA	0.320485	0.399805	0.927147	0.022*	
H2AB	0.157043	0.437020	0.812196	0.022*	
C3A	0.1565 (2)	0.35136 (6)	0.73977 (19)	0.0156 (3)	
H3A	0.178227	0.365690	0.620455	0.019*	
C4A	0.3023 (2)	0.30022 (6)	0.7759 (2)	0.0159 (3)	
H4AA	0.428771	0.301068	0.701986	0.019*	
H4AB	0.350225	0.299338	0.897950	0.019*	
C5A	0.1606 (2)	0.24957 (6)	0.73260 (18)	0.0139 (3)	
H5A	0.145630	0.245438	0.604934	0.017*	
O2A	0.23565 (18)	0.19773 (4)	0.80652 (14)	0.0176 (3)	
H2A	0.341384	0.186208	0.752123	0.026*	
C6A	−0.0529 (2)	0.26608 (6)	0.80698 (19)	0.0144 (3)	
H6AA	−0.051514	0.261229	0.933452	0.017*	
H6AB	−0.169963	0.243131	0.755956	0.017*	
C7A	−0.0780 (2)	0.32858 (6)	0.75773 (18)	0.0141 (3)	
H7A	−0.157303	0.332025	0.645327	0.017*	
C8A	−0.1819 (2)	0.36634 (6)	0.89395 (19)	0.0153 (3)	
H8AA	−0.264574	0.397163	0.838265	0.018*	
H8AB	−0.278621	0.343960	0.966281	0.018*	
O1B	0.5861 (19)	0.6170 (5)	0.6823 (12)	0.0159 (13)	0.463 (2)
H1B	0.498753	0.642917	0.659004	0.024*	0.463 (2)
C1B	0.4722 (14)	0.5665 (4)	0.7261 (9)	0.0135 (13)	0.463 (2)
H1BA	0.406620	0.569882	0.841688	0.016*	0.463 (2)
C2B	0.3060 (7)	0.55080 (17)	0.5881 (6)	0.0158 (8)	0.463 (2)
H2BA	0.223017	0.584327	0.550656	0.019*	0.463 (2)
H2BB	0.206747	0.521799	0.630977	0.019*	0.463 (2)
C3B	0.4388 (6)	0.52750 (15)	0.4400 (5)	0.0160 (8)	0.463 (2)
H3B	0.476345	0.558519	0.358849	0.019*	0.463 (2)
C4B	0.3360 (11)	0.4772 (3)	0.3397 (8)	0.0190 (15)	0.463 (2)
H4BA	0.220722	0.459703	0.406427	0.023*	0.463 (2)
H4BB	0.276787	0.489721	0.227031	0.023*	0.463 (2)
C5B	0.5197 (18)	0.4358 (4)	0.3161 (10)	0.0224 (17)	0.463 (2)
H5B	0.609958	0.448721	0.219222	0.027*	0.463 (2)
O2B	0.4395 (19)	0.3804 (5)	0.2803 (13)	0.0159 (13)	0.463 (2)
H2B	0.540541	0.357204	0.283188	0.024*	0.463 (2)
C6B	0.6454 (9)	0.4400 (3)	0.4836 (9)	0.0144 (10)	0.463 (2)
H6BA	0.792494	0.425919	0.469432	0.017*	0.463 (2)
H6BB	0.576485	0.417681	0.574584	0.017*	0.463 (2)
C7B	0.6468 (6)	0.50277 (15)	0.5295 (5)	0.0150 (7)	0.463 (2)
H7B	0.775856	0.521342	0.481846	0.018*	0.463 (2)
C8B	0.6282 (9)	0.5170 (2)	0.7203 (7)	0.0143 (11)	0.463 (2)
H8BA	0.768645	0.527798	0.770467	0.017*	0.463 (2)
H8BB	0.572815	0.484252	0.784767	0.017*	0.463 (2)
O1C	0.530 (10)	0.605 (2)	0.791 (7)	0.013 (3)	0.037 (2)
H1C	0.438106	0.630366	0.780166	0.019*	0.037 (2)
C1C	0.560 (7)	0.5784 (17)	0.631 (6)	0.016 (2)	0.037 (2)
H1CA	0.635742	0.605584	0.554858	0.019*	0.037 (2)
C2C	0.359 (8)	0.559 (3)	0.536 (11)	0.016 (3)	0.037 (2)
H2CA	0.283622	0.591060	0.480986	0.019*	0.037 (2)
H2CB	0.260712	0.539825	0.615503	0.019*	0.037 (2)
C3C	0.438 (7)	0.5170 (14)	0.399 (6)	0.016 (2)	0.037 (2)
H3C	0.450647	0.536264	0.284838	0.020*	0.037 (2)
C4C	0.302 (6)	0.4632 (16)	0.380 (7)	0.018 (3)	0.037 (2)
H4CA	0.204899	0.459624	0.478460	0.022*	0.037 (2)
H4CB	0.215178	0.464396	0.272447	0.022*	0.037 (2)
C5C	0.457 (7)	0.4142 (14)	0.377 (6)	0.018 (3)	0.037 (2)
H5C	0.394294	0.384475	0.452572	0.022*	0.037 (2)
O2C	0.478 (11)	0.389 (3)	0.215 (8)	0.016 (4)	0.037 (2)
H2C	0.357848	0.377921	0.178129	0.024*	0.037 (2)
C6C	0.657 (9)	0.4329 (17)	0.468 (10)	0.017 (3)	0.037 (2)
H6CA	0.782747	0.417484	0.408304	0.020*	0.037 (2)
H6CB	0.660553	0.418732	0.587501	0.020*	0.037 (2)
C7C	0.666 (6)	0.4971 (16)	0.467 (6)	0.016 (3)	0.037 (2)
H7C	0.778980	0.510540	0.388575	0.019*	0.037 (2)
C8C	0.694 (11)	0.525 (2)	0.646 (7)	0.014 (3)	0.037 (2)
H8CA	0.641416	0.500103	0.737544	0.017*	0.037 (2)
H8CB	0.845499	0.534256	0.670060	0.017*	0.037 (2)

### Atomic displacement parameters (Å^2^)

**Table:**

	*U*^11^	*U*^22^	*U*^33^	*U*^12^	*U*^13^	*U*^23^
O1A	0.0158 (6)	0.0160 (5)	0.0173 (6)	−0.0030 (4)	−0.0062 (4)	0.0011 (4)
C1A	0.0169 (8)	0.0098 (7)	0.0189 (7)	0.0002 (5)	−0.0027 (6)	−0.0011 (5)
C2A	0.0200 (8)	0.0097 (7)	0.0242 (8)	−0.0038 (6)	−0.0005 (6)	0.0022 (6)
C3A	0.0170 (8)	0.0143 (7)	0.0156 (7)	−0.0034 (6)	0.0009 (5)	0.0026 (6)
C4A	0.0124 (7)	0.0159 (8)	0.0193 (7)	−0.0024 (5)	0.0007 (5)	−0.0022 (6)
C5A	0.0144 (7)	0.0132 (7)	0.0140 (7)	−0.0007 (5)	−0.0003 (5)	−0.0006 (5)
O2A	0.0198 (6)	0.0121 (5)	0.0211 (6)	0.0023 (4)	0.0014 (4)	−0.0002 (4)
C6A	0.0127 (7)	0.0126 (7)	0.0179 (7)	−0.0032 (5)	−0.0005 (5)	−0.0019 (6)
C7A	0.0145 (7)	0.0146 (7)	0.0130 (7)	−0.0007 (5)	−0.0039 (5)	0.0005 (5)
C8A	0.0138 (7)	0.0132 (7)	0.0186 (7)	0.0024 (5)	−0.0038 (6)	0.0005 (6)
O1B	0.014 (2)	0.0119 (10)	0.022 (4)	0.0030 (13)	−0.003 (2)	−0.0091 (19)
C1B	0.012 (2)	0.009 (2)	0.019 (3)	0.0011 (15)	−0.008 (2)	−0.004 (2)
C2B	0.0156 (19)	0.0079 (17)	0.024 (2)	−0.0010 (13)	−0.0016 (15)	0.0003 (15)
C3B	0.0218 (16)	0.0037 (16)	0.0220 (18)	−0.0016 (15)	−0.0110 (14)	0.0005 (14)
C4B	0.023 (3)	0.014 (2)	0.020 (3)	0.005 (2)	−0.009 (2)	0.000 (2)
C5B	0.029 (3)	0.012 (2)	0.026 (3)	0.0004 (19)	−0.005 (3)	−0.006 (2)
O2B	0.014 (2)	0.0119 (10)	0.022 (4)	0.0030 (13)	−0.003 (2)	−0.0091 (19)
C6B	0.0140 (19)	0.009 (2)	0.020 (2)	0.0024 (14)	−0.0048 (17)	−0.0043 (18)
C7B	0.0167 (16)	0.0077 (15)	0.0202 (19)	0.0013 (13)	−0.0088 (14)	−0.0041 (15)
C8B	0.020 (2)	0.0069 (18)	0.016 (2)	0.0010 (14)	−0.0075 (19)	−0.0003 (18)
O1C	0.011 (6)	0.010 (6)	0.017 (6)	0.003 (6)	0.000 (6)	−0.008 (6)
C1C	0.016 (4)	0.011 (4)	0.020 (4)	0.001 (4)	−0.006 (4)	−0.003 (4)
C2C	0.017 (5)	0.008 (5)	0.022 (5)	0.002 (5)	−0.004 (5)	−0.001 (5)
C3C	0.019 (4)	0.008 (4)	0.022 (4)	0.004 (4)	−0.006 (4)	0.000 (4)
C4C	0.021 (5)	0.010 (5)	0.023 (5)	0.002 (5)	−0.009 (5)	−0.001 (5)
C5C	0.020 (4)	0.012 (4)	0.023 (5)	0.003 (4)	−0.005 (4)	−0.005 (4)
O2C	0.018 (6)	0.010 (6)	0.020 (6)	0.001 (6)	−0.001 (6)	−0.007 (6)
C6C	0.020 (5)	0.008 (5)	0.022 (5)	0.004 (5)	−0.004 (5)	−0.005 (5)
C7C	0.018 (5)	0.008 (5)	0.021 (5)	0.002 (4)	−0.008 (5)	−0.003 (5)
C8C	0.015 (5)	0.008 (5)	0.020 (5)	0.002 (5)	−0.008 (5)	−0.002 (5)

### Geometric parameters (Å, º)

**Table:**

O1A—H1A	0.8400	C4B—C5B	1.527 (11)
O1A—C1A	1.4365 (18)	C5B—H5B	1.0000
C1A—H1AA	1.0000	C5B—O2B	1.431 (12)
C1A—C2A	1.535 (2)	C5B—C6B	1.511 (9)
C1A—C8A	1.530 (2)	O2B—H2B	0.8400
C2A—H2AA	0.9900	C6B—H6BA	0.9900
C2A—H2AB	0.9900	C6B—H6BB	0.9900
C2A—C3A	1.543 (2)	C6B—C7B	1.529 (6)
C3A—H3A	1.0000	C7B—H7B	1.0000
C3A—C4A	1.540 (2)	C7B—C8B	1.528 (6)
C3A—C7A	1.577 (2)	C8B—H8BA	0.9900
C4A—H4AA	0.9900	C8B—H8BB	0.9900
C4A—H4AB	0.9900	O1C—H1C	0.8400
C4A—C5A	1.527 (2)	O1C—C1C	1.412 (18)
C5A—H5A	1.0000	C1C—H1CA	1.0000
C5A—O2A	1.4316 (18)	C1C—C2C	1.522 (18)
C5A—C6A	1.522 (2)	C1C—C8C	1.521 (18)
O2A—H2A	0.8400	C2C—H2CA	0.9900
C6A—H6AA	0.9900	C2C—H2CB	0.9900
C6A—H6AB	0.9900	C2C—C3C	1.547 (18)
C6A—C7A	1.537 (2)	C3C—H3C	1.0000
C7A—H7A	1.0000	C3C—C4C	1.540 (18)
C7A—C8A	1.542 (2)	C3C—C7C	1.585 (16)
C8A—H8AA	0.9900	C4C—H4CA	0.9900
C8A—H8AB	0.9900	C4C—H4CB	0.9900
O1B—H1B	0.8400	C4C—C5C	1.516 (17)
O1B—C1B	1.438 (12)	C5C—H5C	1.0000
C1B—H1BA	1.0000	C5C—O2C	1.410 (18)
C1B—C2B	1.527 (8)	C5C—C6C	1.496 (17)
C1B—C8B	1.530 (9)	O2C—H2C	0.8400
C2B—H2BA	0.9900	C6C—H6CA	0.9900
C2B—H2BB	0.9900	C6C—H6CB	0.9900
C2B—C3B	1.538 (6)	C6C—C7C	1.522 (17)
C3B—H3B	1.0000	C7C—H7C	1.0000
C3B—C4B	1.557 (7)	C7C—C8C	1.542 (18)
C3B—C7B	1.579 (4)	C8C—H8CA	0.9900
C4B—H4BA	0.9900	C8C—H8CB	0.9900
C4B—H4BB	0.9900		
			
C1A—O1A—H1A	109.5	C4B—C5B—H5B	109.6
O1A—C1A—H1AA	110.9	O2B—C5B—C4B	110.4 (9)
O1A—C1A—C2A	112.09 (12)	O2B—C5B—H5B	109.6
O1A—C1A—C8A	108.43 (11)	O2B—C5B—C6B	113.8 (9)
C2A—C1A—H1AA	110.9	C6B—C5B—C4B	103.7 (5)
C8A—C1A—H1AA	110.9	C6B—C5B—H5B	109.6
C8A—C1A—C2A	103.49 (12)	C5B—O2B—H2B	109.5
C1A—C2A—H2AA	110.6	C5B—C6B—H6BA	110.7
C1A—C2A—H2AB	110.6	C5B—C6B—H6BB	110.7
C1A—C2A—C3A	105.75 (12)	C5B—C6B—C7B	105.4 (5)
H2AA—C2A—H2AB	108.7	H6BA—C6B—H6BB	108.8
C3A—C2A—H2AA	110.6	C7B—C6B—H6BA	110.7
C3A—C2A—H2AB	110.6	C7B—C6B—H6BB	110.7
C2A—C3A—H3A	110.1	C3B—C7B—H7B	110.0
C2A—C3A—C7A	105.45 (12)	C6B—C7B—C3B	104.9 (3)
C4A—C3A—C2A	115.28 (12)	C6B—C7B—H7B	110.0
C4A—C3A—H3A	110.1	C8B—C7B—C3B	105.5 (3)
C4A—C3A—C7A	105.45 (11)	C8B—C7B—C6B	116.1 (4)
C7A—C3A—H3A	110.1	C8B—C7B—H7B	110.0
C3A—C4A—H4AA	111.0	C1B—C8B—H8BA	110.7
C3A—C4A—H4AB	111.0	C1B—C8B—H8BB	110.7
H4AA—C4A—H4AB	109.0	C7B—C8B—C1B	105.0 (4)
C5A—C4A—C3A	103.72 (12)	C7B—C8B—H8BA	110.7
C5A—C4A—H4AA	111.0	C7B—C8B—H8BB	110.7
C5A—C4A—H4AB	111.0	H8BA—C8B—H8BB	108.8
C4A—C5A—H5A	109.7	C1C—O1C—H1C	109.5
O2A—C5A—C4A	113.61 (12)	O1C—C1C—H1CA	108.1
O2A—C5A—H5A	109.7	O1C—C1C—C2C	116 (3)
O2A—C5A—C6A	110.66 (12)	O1C—C1C—C8C	113 (2)
C6A—C5A—C4A	103.16 (11)	C2C—C1C—H1CA	108.1
C6A—C5A—H5A	109.7	C8C—C1C—H1CA	108.1
C5A—O2A—H2A	109.5	C8C—C1C—C2C	104 (2)
C5A—C6A—H6AA	111.0	C1C—C2C—H2CA	110.8
C5A—C6A—H6AB	111.0	C1C—C2C—H2CB	110.8
C5A—C6A—C7A	103.84 (12)	C1C—C2C—C3C	104.6 (18)
H6AA—C6A—H6AB	109.0	H2CA—C2C—H2CB	108.9
C7A—C6A—H6AA	111.0	C3C—C2C—H2CA	110.8
C7A—C6A—H6AB	111.0	C3C—C2C—H2CB	110.8
C3A—C7A—H7A	110.1	C2C—C3C—H3C	110.4
C6A—C7A—C3A	105.14 (11)	C2C—C3C—C7C	105.0 (16)
C6A—C7A—H7A	110.1	C4C—C3C—C2C	114 (3)
C6A—C7A—C8A	115.49 (12)	C4C—C3C—H3C	110.4
C8A—C7A—C3A	105.76 (11)	C4C—C3C—C7C	106.3 (15)
C8A—C7A—H7A	110.1	C7C—C3C—H3C	110.4
C1A—C8A—C7A	105.40 (12)	C3C—C4C—H4CA	110.5
C1A—C8A—H8AA	110.7	C3C—C4C—H4CB	110.5
C1A—C8A—H8AB	110.7	H4CA—C4C—H4CB	108.7
C7A—C8A—H8AA	110.7	C5C—C4C—C3C	106.2 (16)
C7A—C8A—H8AB	110.7	C5C—C4C—H4CA	110.5
H8AA—C8A—H8AB	108.8	C5C—C4C—H4CB	110.5
C1B—O1B—H1B	109.5	C4C—C5C—H5C	105.7
O1B—C1B—H1BA	111.3	O2C—C5C—C4C	114 (2)
O1B—C1B—C2B	111.9 (8)	O2C—C5C—H5C	105.7
O1B—C1B—C8B	108.1 (8)	O2C—C5C—C6C	117 (3)
C2B—C1B—H1BA	111.3	C6C—C5C—C4C	107.4 (17)
C2B—C1B—C8B	102.7 (5)	C6C—C5C—H5C	105.7
C8B—C1B—H1BA	111.3	C5C—O2C—H2C	109.5
C1B—C2B—H2BA	111.0	C5C—C6C—H6CA	109.9
C1B—C2B—H2BB	111.0	C5C—C6C—H6CB	109.9
C1B—C2B—C3B	103.9 (4)	C5C—C6C—C7C	108.9 (17)
H2BA—C2B—H2BB	109.0	H6CA—C6C—H6CB	108.3
C3B—C2B—H2BA	111.0	C7C—C6C—H6CA	109.9
C3B—C2B—H2BB	111.0	C7C—C6C—H6CB	109.9
C2B—C3B—H3B	110.3	C3C—C7C—H7C	110.1
C2B—C3B—C4B	115.1 (4)	C6C—C7C—C3C	105.5 (15)
C2B—C3B—C7B	105.0 (3)	C6C—C7C—H7C	110.1
C4B—C3B—H3B	110.3	C6C—C7C—C8C	116 (3)
C4B—C3B—C7B	105.5 (3)	C8C—C7C—C3C	105.1 (16)
C7B—C3B—H3B	110.3	C8C—C7C—H7C	110.1
C3B—C4B—H4BA	110.9	C1C—C8C—C7C	103.6 (18)
C3B—C4B—H4BB	110.9	C1C—C8C—H8CA	111.0
H4BA—C4B—H4BB	108.9	C1C—C8C—H8CB	111.0
C5B—C4B—C3B	104.2 (6)	C7C—C8C—H8CA	111.0
C5B—C4B—H4BA	110.9	C7C—C8C—H8CB	111.0
C5B—C4B—H4BB	110.9	H8CA—C8C—H8CB	109.0
			
O1A—C1A—C2A—C3A	80.65 (15)	C3B—C7B—C8B—C1B	−21.7 (5)
O1A—C1A—C8A—C7A	−82.34 (14)	C4B—C3B—C7B—C6B	−2.7 (5)
C1A—C2A—C3A—C4A	−94.67 (15)	C4B—C3B—C7B—C8B	−125.9 (4)
C1A—C2A—C3A—C7A	21.20 (15)	C4B—C5B—C6B—C7B	−40.4 (9)
C2A—C1A—C8A—C7A	36.83 (14)	C5B—C6B—C7B—C3B	26.3 (7)
C2A—C3A—C4A—C5A	140.20 (13)	C5B—C6B—C7B—C8B	142.3 (6)
C2A—C3A—C7A—C6A	−121.19 (12)	O2B—C5B—C6B—C7B	−160.3 (8)
C2A—C3A—C7A—C8A	1.47 (15)	C6B—C7B—C8B—C1B	−137.4 (5)
C3A—C4A—C5A—O2A	−161.11 (12)	C7B—C3B—C4B—C5B	−21.4 (7)
C3A—C4A—C5A—C6A	−41.26 (14)	C8B—C1B—C2B—C3B	−41.7 (6)
C3A—C7A—C8A—C1A	−23.66 (14)	O1C—C1C—C2C—C3C	−163 (4)
C4A—C3A—C7A—C6A	1.22 (14)	O1C—C1C—C8C—C7C	168 (4)
C4A—C3A—C7A—C8A	123.88 (12)	C1C—C2C—C3C—C4C	137 (5)
C4A—C5A—C6A—C7A	42.17 (14)	C1C—C2C—C3C—C7C	21 (5)
C5A—C6A—C7A—C3A	−26.43 (14)	C2C—C1C—C8C—C7C	42 (5)
C5A—C6A—C7A—C8A	−142.58 (12)	C2C—C3C—C4C—C5C	−134 (4)
O2A—C5A—C6A—C7A	164.03 (11)	C2C—C3C—C7C—C6C	127 (5)
C6A—C7A—C8A—C1A	92.14 (14)	C2C—C3C—C7C—C8C	4 (5)
C7A—C3A—C4A—C5A	24.33 (14)	C3C—C4C—C5C—O2C	−107 (4)
C8A—C1A—C2A—C3A	−35.97 (14)	C3C—C4C—C5C—C6C	24 (5)
O1B—C1B—C2B—C3B	74.0 (7)	C3C—C7C—C8C—C1C	−28 (5)
O1B—C1B—C8B—C7B	−79.0 (7)	C4C—C3C—C7C—C6C	6 (5)
C1B—C2B—C3B—C4B	143.5 (5)	C4C—C3C—C7C—C8C	−117 (5)
C1B—C2B—C3B—C7B	28.0 (5)	C4C—C5C—C6C—C7C	−21 (6)
C2B—C1B—C8B—C7B	39.4 (6)	C5C—C6C—C7C—C3C	9 (6)
C2B—C3B—C4B—C5B	−136.7 (5)	C5C—C6C—C7C—C8C	125 (5)
C2B—C3B—C7B—C6B	119.3 (4)	O2C—C5C—C6C—C7C	109 (5)
C2B—C3B—C7B—C8B	−3.9 (4)	C6C—C7C—C8C—C1C	−144 (4)
C3B—C4B—C5B—O2B	160.1 (7)	C7C—C3C—C4C—C5C	−18 (4)
C3B—C4B—C5B—C6B	38.0 (9)	C8C—C1C—C2C—C3C	−40 (5)

### Hydrogen-bond geometry (Å, º)

**Table:**

*D*—H···*A*	*D*—H	H···*A*	*D*···*A*	*D*—H···*A*
O1*A*—H1*A*···O1*B*^i^	0.84	1.89	2.732 (12)	177
O1*A*—H1*A*···O2*B*^ii^	0.84	1.88	2.710 (12)	170
O1*A*—H1*A*···O1*C*^i^	0.84	2.05	2.83 (6)	154
O1*A*—H1*A*···O2*C*^ii^	0.84	2.04	2.82 (7)	155
C2*A*—H2*AA*···O1*C*^i^	0.99	2.37	3.18 (5)	139
C2*A*—H2*AA*···O2*C*^ii^	0.99	2.44	3.25 (6)	139
O2*A*—H2*A*···O1*A*^iii^	0.84	1.91	2.7432 (16)	173
O1*B*—H1*B*···O2*A*^iv^	0.84	1.98	2.784 (12)	159
O2*B*—H2*B*···O2*A*^iii^	0.84	1.79	2.629 (12)	173
O1*C*—H1*C*···O2*A*^iv^	0.84	2.04	2.86 (5)	164

Symmetry codes: (i) −*x*+1, −*y*+1, −*z*+2; (ii) *x*, *y*, *z*+1; (iii) *x*+1/2, −*y*+1/2, *z*−1/2; (iv) −*x*+1/2, *y*+1/2, −*z*+3/2.
